# Differential expression and regulation of the non-integrin 37/67-kDa laminin receptor on peripheral blood leukocytes of healthy individuals and patients with rheumatoid arthritis

**DOI:** 10.1038/s41598-018-37907-7

**Published:** 2019-02-04

**Authors:** Barry A. Kane, Hongyan An, Poornima Rajasekariah, H. Patrick McNeil, Katherine Bryant, Nicodemus Tedla

**Affiliations:** 1 Mechanisms of Diseases and Translational Research, School of Medical Sciences, Department of Pathology, University of New South Wales, Sydney, Australia; 2 Faculty of Medicine and Health Sciences, Macquarie University, New South Wales, Australia; 3 South Western Sydney Clinical School, University of New South Wales, Sydney, Australia

## Abstract

The non-integrin 37/67-kDa laminin receptor (LAMR1) is a complex protein with diverse functions. LAMR1 is widely expressed in epithelial cells and recently it was reported on neutrophils and a subset of activated T cells. Ligation of LAMR1 on peripheral blood mononuclear cells (PBMC) downregulated LPS-induced TNFα production, suggesting immune functions. However, its expression on primary monocytes remain unknown. Interestingly, LAMR1 mRNA is downregulated in PBMC of patients with early rheumatoid arthritis (RA), and low gene expression is an independent predictor of poor response to anti-TNFα treatment, suggesting a role in RA pathogenesis. We found LAMR1 was constitutively expressed on all peripheral blood monocytes and a subset of B cells from healthy individuals and patients with RA and it was abundantly present in synovial tissue of patients with RA. On monocytes and synovial tissue lower levels of LAMR1 expression tended to correlate with increased disease activity scores. *In vitro* treatment of monocytes with IFNγ or IL-10 up-regulated surface LAMR1 in healthy individuals and patients with RA with greater effects observed in healthy individuals. Importantly, treatment with IFNγ significantly increased specific binding of monocytes to laminin-1. TNFα and IL-1β caused marginal downregulation of LAMR1 in patients but effects in controls were variable. Taken together, constitutively expressed LAMR1 on monocytes is differentially regulated by pro-inflammatory and immune-regulatory cytokines suggesting LAMR1 may regulate the threshold and amplitude of their activation and migration. Decreased levels in patients with RA may indicate loss of this potentially critical homeostatic regulation thereby contributing to the excessive inflammation.

## Introduction

Laminins are a family of large glycoproteins, typically found within the basal lamina of the extracellular matrix^1–3^. Currently there are 15 laminin proteins with crucial and diverse roles including cell adhesion, migration, proliferation, differentiation and cellular communication with the extracellular environment^1–3^. Laminins are believed to exert these complex regulatory effects through interaction with their receptors. One of such receptors is the non-integrin 37/67-kDa laminin receptor (aka laminin receptor 1 or LAMR1)^4^. LAMR1, originally identified on various epithelial malignancies, is structurally complex protein found on the cell surface, in the cytosol, in the nucleus and in association with the ribosomes^4–6^. LAMR1 exerts diverse functions that extends far beyond cell adhesion via laminins to include ribosomal biogenesis and translation, pre-ribosomal RNA processing, cell migration, invasion, viability and growth, cytoskeletal reorganisation as well as binding to histones and chromatin^4^. LAMR1 is believed to play key roles in a wide range of diseases including tumour growth and metastasis^4,7,8^, neurotropic infections^9^, prion disease^10^, neurodegeneration^11^ and cellular ageing^12^. Surface LAMR1 is expressed on bone marrow and peripheral blood CD34+ cells and facilitates homing of these cells to the bone marrow^13^. It is also expressed on human neutrophils, a monocytic cell line (U937)^14^ and a subset of activated T cells^15^. *In vitro* data revealed that ligation of LAMR1 on PBMCs with a pharmacological ligand, epigallocatechin-3-gallate (EGCG), down-regulated toll-like receptor 2 and 4 mediated responses^16,17^. These results suggest that LAMR1 may have immune regulatory functions and its altered expression may contribute to unregulated excessive inflammation in chronic diseases such as rheumatoid arthritis (RA).

RA is a systemic autoimmune disease characterised by excessive chronic joint inflammation^18–20^. Laminin-1 produced by synovial fibroblasts and endothelial cells of patients with RA^2,21^ promotes adhesion and migration of the inflammatory cells^22,23^. Interestingly, studies revealed that LAMR1 negatively regulates GM-CSF receptor signalling in neutrophils^24^, an important target in the treatment of RA^25^. Moreover, LAMR1 gene is downregulated in peripheral blood mononuclear cells (PBMCs) of patients with early RA compared to those with established disease^26^, and low LAMR1 mRNA expression in PBMCs was found to be an independent predictor of poor response to anti-TNF-α therapy^27^. These observations suggest that LAMR1 may play a key role in the pathogenesis of RA. The aim of this study was to determine the expression, regulation and function of LAMR1 on peripheral blood leukocytes of healthy individuals and patients with RA and assess its expression in synovial tissue. We found that LAMR1 is expressed on the surface of peripheral blood monocytes of healthy individuals and patients with RA its expression in patients negatively correlated with disease activity. We also discovered that LAMR1 is highly expressed in synovial tissue of patients with RA and is tightly regulated by cytokines relevant to the pathogenesis of RA.

## Results

### Expression of LAMR1 on PBMCs of healthy individuals and patients with RA

**Figure 1:**
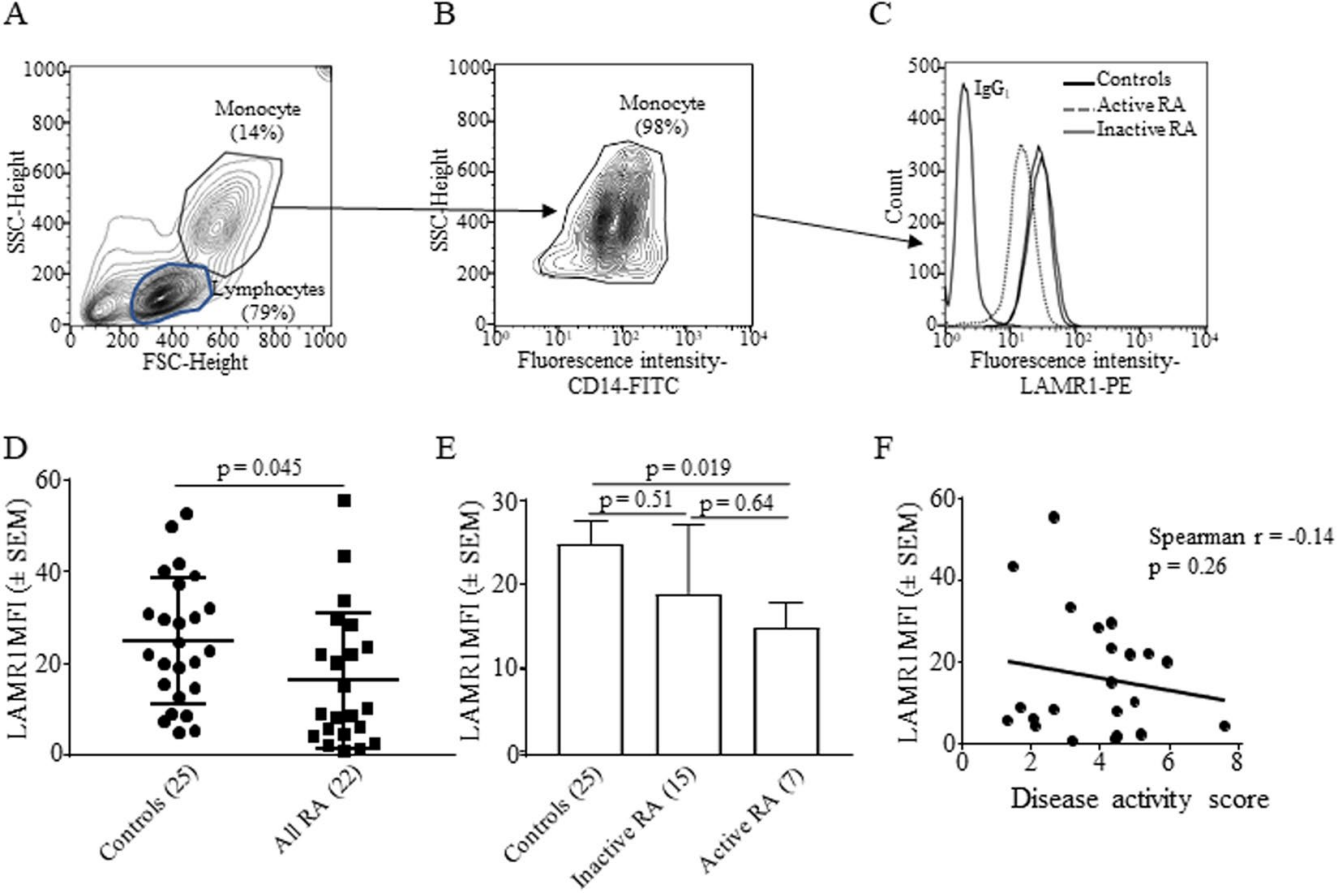
Representative Flow cytometric analysis of LAMR1 expression on peripheral blood monocytes gated from density gradient separated blood mononuclear cells based on their side and forward scatter **(A)** and sub-gated based on surface expression of CD14 (**B**) and mean fluorescent intensity (MFI) of surface LAMR1 determined by histogram plots of the gated monocyte populations. (**C**) The average MFI of LAMR1 expression on monocytes of patients with RA was significantly lower than healthy control individuals. (**D**) Further stratification of patients showed that monocytes derived from patients with active RA, had the lowest surface LAMR1 expression levels when compared to patients with inactive RA and control individuals. (**E**) Linear regression analysis and Spearman’s correlation study in patients with RA showed negative correlation between LAMR1 expression levels on monocytes and disease activity score but this was not statistically significant (**F**).

**Figure 2:**
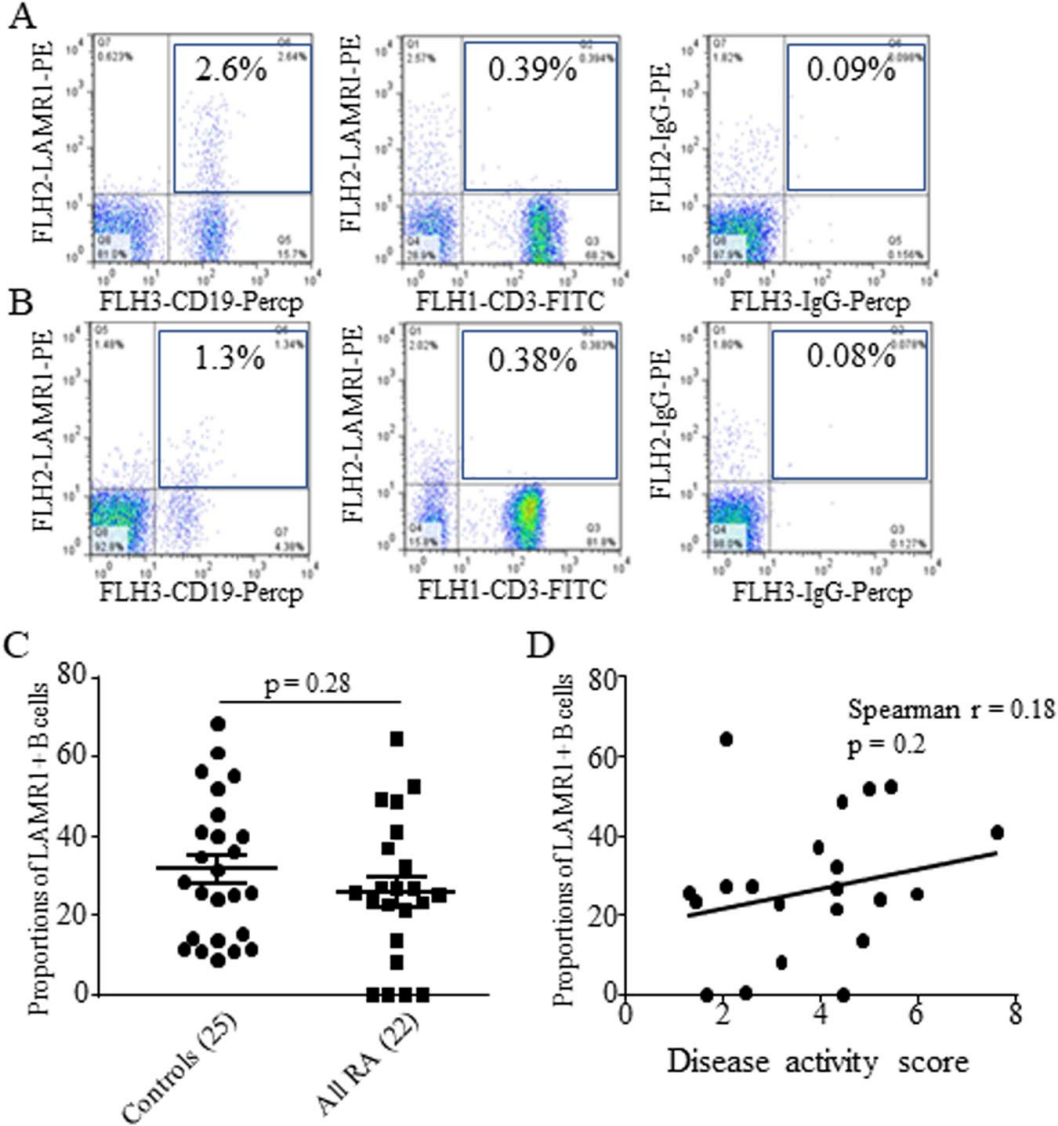
Representative flow cytometric analysis of LAMR1 expression on peripheral blood CD19+ B and CD3+ T cells of a healthy subject (**A**) and a patient with active RA; (**B**) upper right quadrant of each plot show proportions of LAMR1+ve B and T cells, relative to isotype and fluorochrome-matched negative control Abs stained cells. The mean proportions (±SEM) of LAMR1 expressing B cells in patients with RA was lower than healthy control individuals but this was not statistically significant. (**C**) Although not statistically significant, linear regression analysis in patients with RA showed positive correlation between proportions of LAMR1+ B cells and disease activity score (**D**).

Flow cytometric analysis on monocytes gated from peripheral blood mononuclear cells based on their forward and side scatter profile (Fig. 1A) and surface CD14 expression (Fig. 1B) showed constitutive expression of LAMR1 in 100% of monocytes from healthy individuals and patients with RA regardless of their disease activity (Fig. 1C). The mean fluorescence intensity (MFI ± SEM) of LAMR1 expression on monocytes from patients with RA was 16.3 ± 3.2 and this was significantly lower than the average MFI of 24.8 ± 2.7 observed on monocytes of healthy individuals (p = 0.045) (Fig. 1D). The average MFI of LAMR1 on monocytes obtained from patients with active disease (14.9 ± 2.9) was measurably lower than those with inactive disease (19.0 ± 8.0), although not statistically significant (p = 0.64; Fig. 1E). However, LAMR1 expression on monocytes of patients with active disease was significantly lower than healthy individuals (p = 0.019; Fig. 1E). In patients with RA lower levels of LAMR1 expression on monocytes tended to correlate with increased disease activity scores (Spearman r = −0.14, p = 0.26) (Fig. 1F). Surface expression of LAMR1 was also detected in a small subset of peripheral blood B cells in healthy individuals (Fig. 2A) (average 2.3 ± 0.9%) and patients with RA (Fig. 2B) (average 1.4 ± 1.1%). Like LAMR1 expression on monocytes, the proportions of LAMR1 expressing B cells in healthy individuals were measurably higher (31.5 ± 3.5% of all B cells) than those found in patients with RA (25.9 ± 3.8% of all B cells), although this was not statistically significant (p = 0.28; Fig. 2C). There was a positive but not statistically significant correlation between % of LAMR1+ B cells and increased disease activity (Fig. 2D). Little or no LAMR1 was expressed on T cells (Fig. 2A,B) and NK cells of healthy individuals or patients with RA.

### Regulation of LAMR1 expression on monocytes of healthy individuals and patients with RA by immune-regulatory and pro-inflammatory cytokines *in vitro*

**Figure 3:**
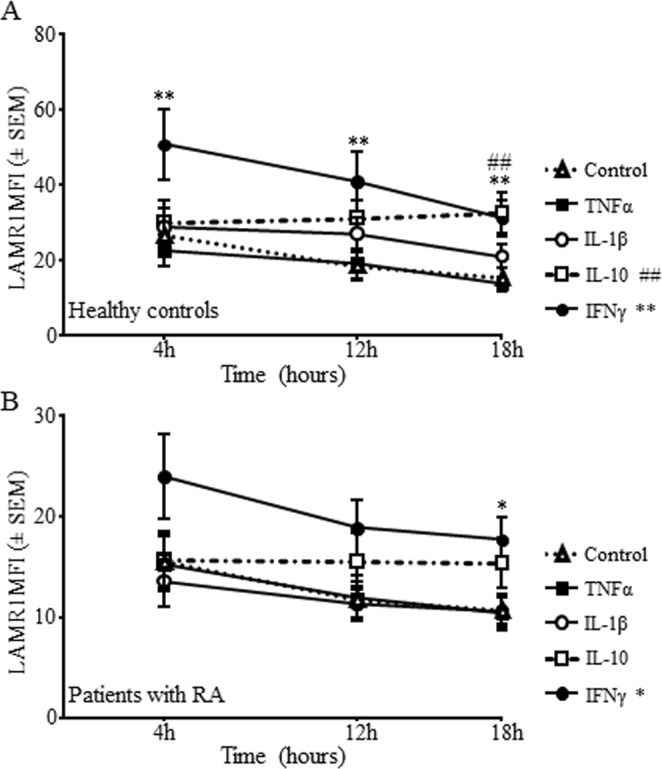
*In vitro* treatment of peripheral blood monocytes from healthy individuals with recombinant cytokines for 4 h, 12 h or 18 h showed significant early sustained upregulation of LAMR1 in response to IFNγ and a delayed increase in response to IL-10 but TNFα and IL-1β caused marginally variable effects. (**A**) Similar responses were observed on monocytes derived from patients with active RA albeit having lower levels of LAMR1 expression across the board (**B**) (n = 5/group; *p < 0.05, **p < 0.01, ^##^p < 0.01 compared to control cells cultured in media alone).

**Figure 4:**
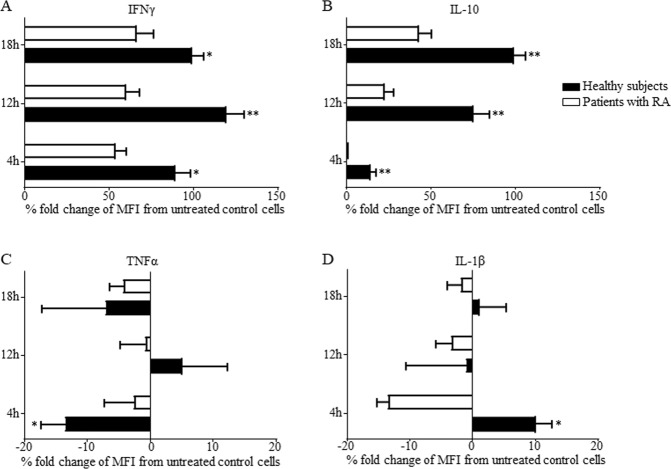
Early and sustained fold increase in MFI (±SEM) of LAMR1 on monocytes of healthy individuals in response to IFNγ was significantly higher than responses to IFNγ by monocytes derived from patients with RA. (**A**) Progressive fold increase in MFI of LAMR1 following IL-10 treatment of monocytes of healthy individuals was significantly higher than the increase observed in patients with RA. (**B**) Note that the magnitude of upregulation of LAMR1 in response to IFNγ was markedly higher than the effects of IL-10 treatment in both healthy individuals and patients. Treatment with TNFα caused modest suppression of LAMR1 on monocytes of healthy individuals and patients with RA at early (4 h) and late (18 h) timepoints, and slightly enhanced expression at 12 h only in healthy individuals. (**C**) Responses to IL-1β by monocytes derived from both healthy individuals and patients were modest, although the effects on monocytes from patients were suppressive at all timepoints while effects on healthy individuals were time-dependent (**D**).

Treatment of PBMCs with recombinant IFNγ or IL-10 *in vitro* caused upregulation of LAMR1 on monocytes of healthy individuals and patients with active RA in a time dependent manner (Fig. 3). The increase in LAMR1 expression in response to IFNγ was rapid starting at 4 h (Fig. 3A) and was sustained for 18 h (Fig. 3B,C) in both healthy controls and patients while responses to IL-10 were slow and gradual with peak effects at 12 h for controls and 18 h for patients (Fig. 3). In contrast, treatment with recombinant TNFα generally caused modest suppression of LAMR1 surface expression in both controls and patients at all timepoints (Fig. 3A–C), except for a marginal up-regulation in controls individuals at 12 h timepoint (Fig. 3B). Although marginal, effects of IL-1β on monocytes derived from patients were suppressive while there was little or no effects on monocytes from healthy individuals at all timepoints (Fig. 3A–C). The fold increase in mean fluorescent intensity (MFI ± SEM) of LAMR1 on monocytes of healthy individuals in response to IFNγ compared to their corresponding untreated control cells at 4 h, 12 h and 18 h timepoints was 89.9 ± 8.7%, 119.8 ± 10.7% and 101.1 ± 6.8% respectively (Fig. 4A). These marked upregulations in healthy individuals were significantly higher than responses to IFNγ by monocytes derived from patients with RA that showed increases by 54.3 ± 6.4%, 60.5 ± 8.2% and 66.8 ± 9.8% respectively (Fig. 4A). The average increase in MFI of LAMR1 following IL-10 treatment on monocytes of healthy individuals were 14.1 ± 3.3%, 75.4 ± 9.7% and 99.5 ± 7.1% at 4, 12 and 18 h timepoints respectively (Fig. 4B), and these were significantly higher than the 1.5 ± 3.7%, 22.8 ± 5.1% and 43.2 ± 7.0% increase observed in patients with RA (Fig. 4B). Interestingly, treatment with TNFα caused suppression of LAMR1 on monocytes of healthy individuals at early (4 h) and late (18 h) timepoints by modest 13.7 ± 4.0% and 7.0 ± 10.2% respectively, and slightly enhanced expression by 5.2 ± 7.2% at 12 h while causing suppression of LAMR1 expression on monocytes derived from patients at all timepoints (Fig. 4C). Responses to IL-1β by monocytes derived from both healthy individuals and patients were modest, although the effects on monocytes from patients were suppressive at all time points tested while the effects on the healthy individuals were time-dependent (Fig. 4D). Note that the magnitude of upregulation of LAMR1 in response to IFNγ was markedly higher than the effects of IL-10 treatment in both healthy individuals and patients (Fig. 4A,B), while the effects of the proinflammatory cytokines, TNFα and IL-1β were modest (Fig. 4C,D).

### Effects of cytokines on LAMR1mRNA expression in monocytes of healthy individuals *in vitro*

**Figure 5:**
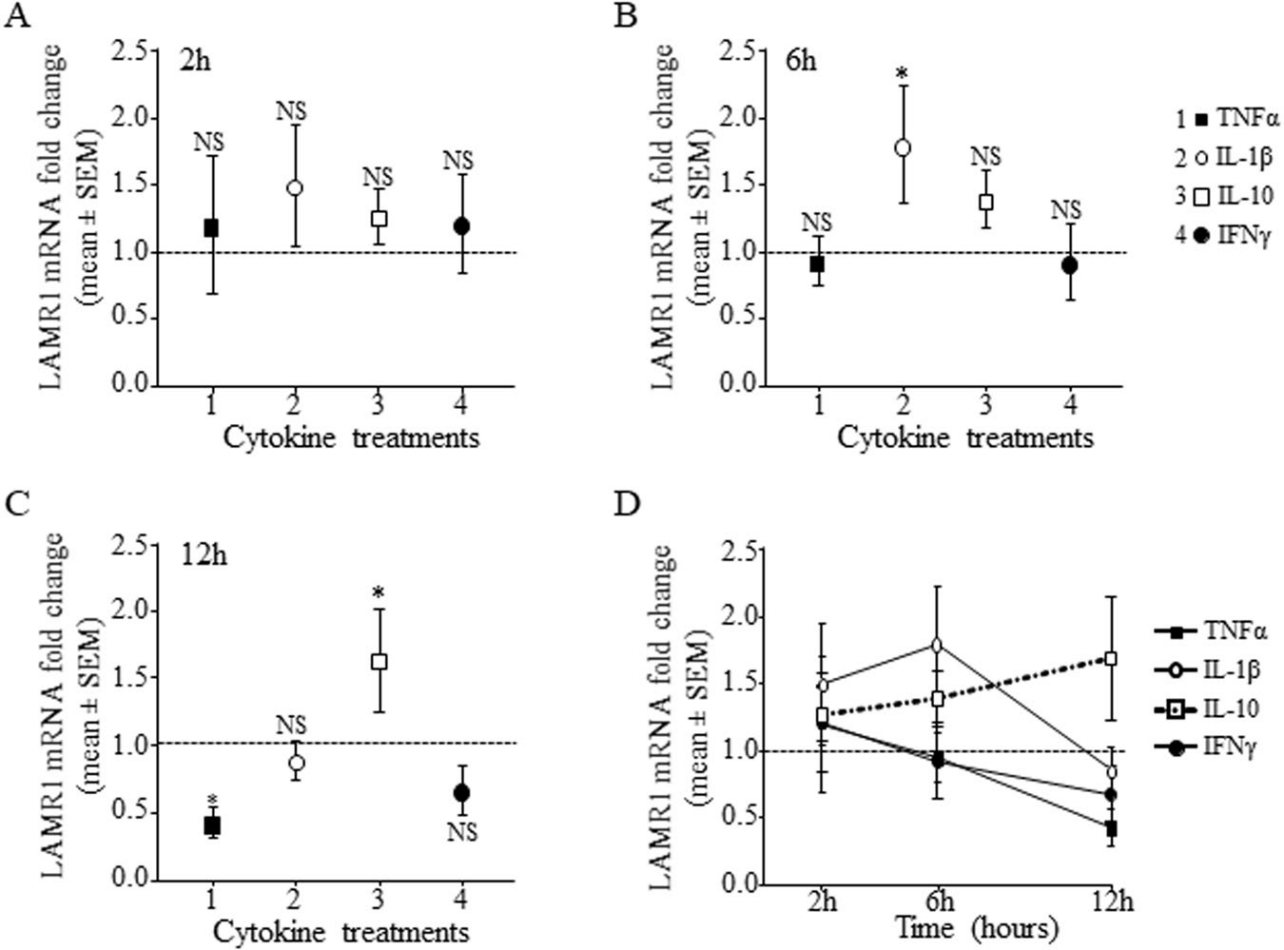
Expression of LAMR1 mRNA in purified monocytes from healthy individuals treated with recombinant cytokines *in vitro* for 2 h (**A**), 6 h (**B**) or 12 h (**C**) presented as fold change from control cells cultured in media alone (dotted horizontal lines) showed significant but transient upregulation by IL-1β, a significant delayed upregulation by IL-10, a significant delayed downregulation by TNFα and little or no effect in response to IFNγ (n = 5; *p < 0.05 when compared to control cells). Summary of the time-dependent effects of recombinant TNFα, IL-1β, IL-10 and IFNγ in LAMR1 mRNA expression (**D**).

**Figure 6:**
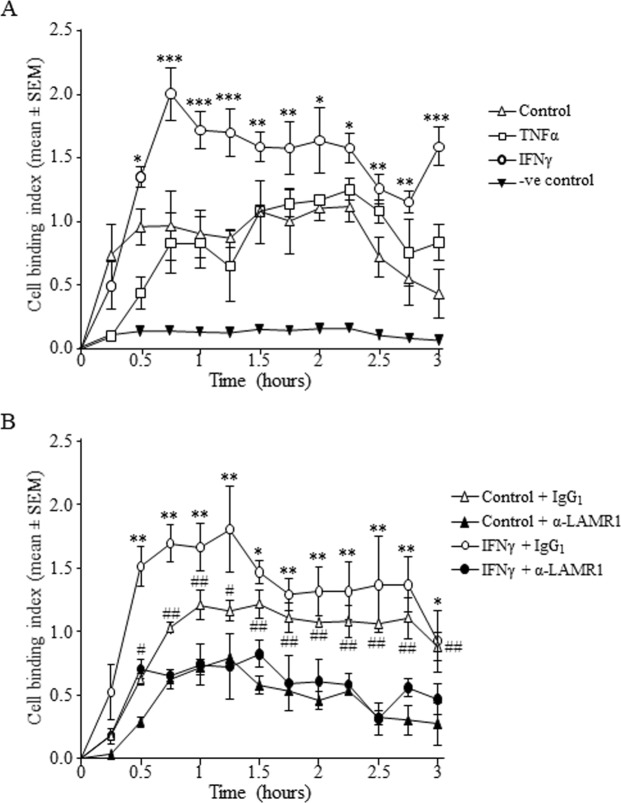
Seeding of purified primary monocytes treated with or without recombinant IFNγ or TNFα showed increased adhesion to laminin-1 coated wells when compared to BSA control coated wells. (**A**) Interestingly, treatment with IFNγ caused significant increase in adhesion to laminin-1 when compared to media alone treated control cells (n = 3; ***p < 0.001, **p < 0.01, *p < 0.05). By contrast, TNFα treated cells showed similar adhesion patterns with controls cells with non-significant differences. Blocking of cell surface LAMR1 on purified primary monocytes treated with or without recombinant IFNγ using specific anti-LAMR1 antibody significantly abrogated binding to plate coated lamini-1 when compared with their corresponding control IgG_1_ treated cells (**B**) (n = 3; **p < 0.01, *p < 0.05 when comparing IFNγ treated cells + α-LAMR1 versus IFNγ treated cells + IgG_1_; ^##^p < 0.01, ^#^p < 0.05 when comparing media alone treated cells + α-LAMR1 versus media alone treated cells + IgG_1_).

Treatment with IFNγ had no effect on mRNA expression of LAMR1 in purified monocytes from healthy individuals after 2 h (Figs. 5A) or 6h (Fig. 5B) treatment and caused marginal decrease after 12 h incubation (Fig. 5C,D), indicating the rapid up-regulation of LAMR1 protein observed in response to IFNγ was likely due to surface mobilisation of pre-formed LAMR1 protein (Fig. 4A). IL-10 caused a significant delayed upregulation of LAMR1 mRNA at the 12 h timepoint (Fig. 5C) and this positive effects in mRNA agreed to its effects on LAMR1 protein expression (Fig. 4B). IL-1β caused a transient significant increase in mRNA at the 6 h timepoint (Fig. 5B) and TNFα significantly downregulated expression at the 12 h timepoints (Fig. 5C).

### Modulation of LAMR1-mediated adhesion of monocyte to laminin-1 by cytokines *in vitro*

Seeding of purified monocytes onto laminin-1-coated wells caused a mean cell binding index (CI) of 0.74 ± 0.4, 0.95 ± 0.1 and 0.96 ± 0.2 after 15, 30 and 45 min incubation respectively, and increased to a maximum of 1.11 ± 0.1 after 2 h (Fig. 6A). Treatment with IFNγ caused an early and sustained increased binding to laminin 1 with mean CI of 1.35 ± 0.04 after 30 min incubation and increased to a maximum of 2.05 ± 0.2 within 45 min and these were an average increase of 30% and 46% respectively when compared to their corresponding untreated control cells (Fig. 6A). Interestingly, treatment with TNFα minimally affected cell binding to laminin-1 when compared to untreated control cells (Fig. 6A). The maximum mean CI of cells seeded onto negative control protein (1% BSA in PBS) was 0.16 ± 0.01 after 2 h incubation which was 7.3 times lower than cells seeded onto laminin-1 coated wells (Fig. 6A), indicating most binding in the experimental samples was via laminin-1.

To assess whether adhesion of the monocytes to laminin-1 was via interaction with LAMR1, cells were pre-incubated with specific mouse anti-LAMR1 monoclonal IgG_1_ or isotype matched irrelevant (-ve) control antibody and seeded onto laminin-1 coated wells. The mean CI of control IgG_1_ treated cells were 0.18 ± 0.06, 0.62 ± 0.04, 1.03 ± 0.08 and 1.21 ± 0.1 after 15, 30, 45 and 60 min incubation respectively (Fig. 6B). By contrast, blocking of LAMR1 with the specific antibody consistently abrogated cell adhesion by an average of 80% after 15 min, by 54% after 30 min, by 40% at 45 min timepoint and by 41% at 60 min timepoint (Fig. 6B). These indicate that early adhesion of monocytes to laminin-1 was predominantly via LAMR1, and LAMR1 contributed to approximately 50% of the adhesion in the later timepoints. Similarly, the increased adhesion of IFNγ-treated cells was markedly and consistently abrogated by 65%, 53%, 61% and 56% after 15, 30, 45 and 60 min incubation respectively when LAMR1 was blocked using specific antibody (Fig. 6B).

### Expression of LAMR1 protein in synovial tissue of patients with RA, degenerative arthritis and traumatic meniscus injury

**Figure 7:**
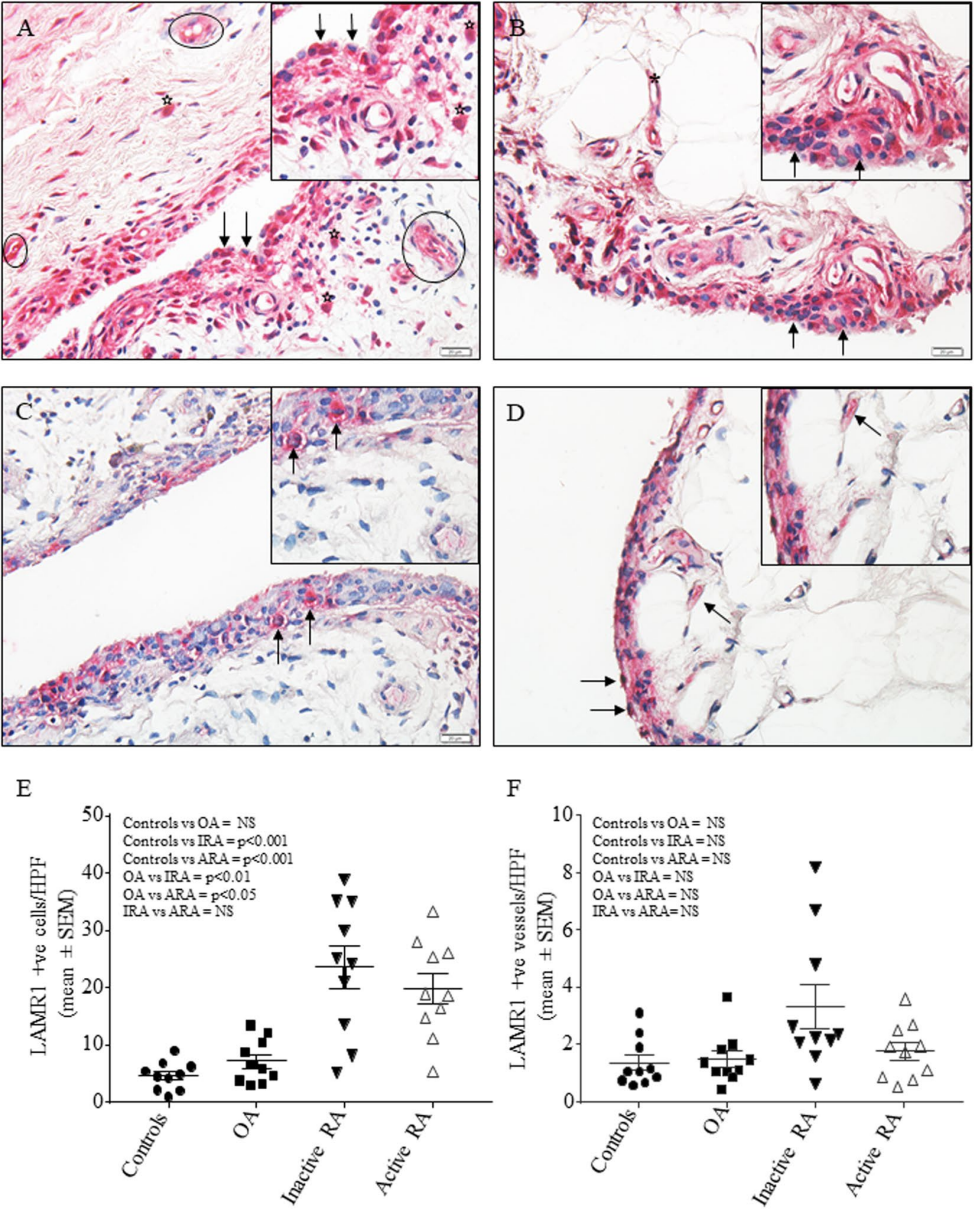
Representative immunohistochemical staining of synovial tissue sections from a patient with active (DAS 4.5) (**A**) and inactive RA (DAS 1.8) (**B**) showing abundant LAMR1 expression by a wide-range of cells including synovial lining cells (arrows), macrophages (stars) and blood vessels (circles) (n = 10/group). In contrast, limited expression of LAMR1 in tissue obtained from patients with OA (**C**) or control individuals (**D**) were primarily restricted to the synovial lining cells (arrows) (n = 10 for each group). Red is positive staining with blue hematoxylin counterstain, imaged using DP73 Olympus bright field microscope (×250 magnification; Insets ×400 magnification). Isotype-matched negative controls showed no non-specific staining in all samples (not shown). Semi-quantitative analysis showing significant increase in the number of LAMR1 +ve cells in patients with active and inactive RA when compared to patients with OA or control individuals. (**E**) Although, patients with tched negative controls showed of LAMR1 +ve cells than patients with active disease (23.6 ± 3.7 versus 19.8 ± 2.6), this was not statistically significant. Similarly, patients with OA had marginally higher numbers of LAMR1 +ve cells when compared to control individuals (n=10/group). Number of LAMR1 +ve blood vessels in synovial tissue of patients with inactive RA were higher than those of patients with active RA, OA and control individuals but none was statistically significant (**F**) (n = 10/group).

**Figure 8:**
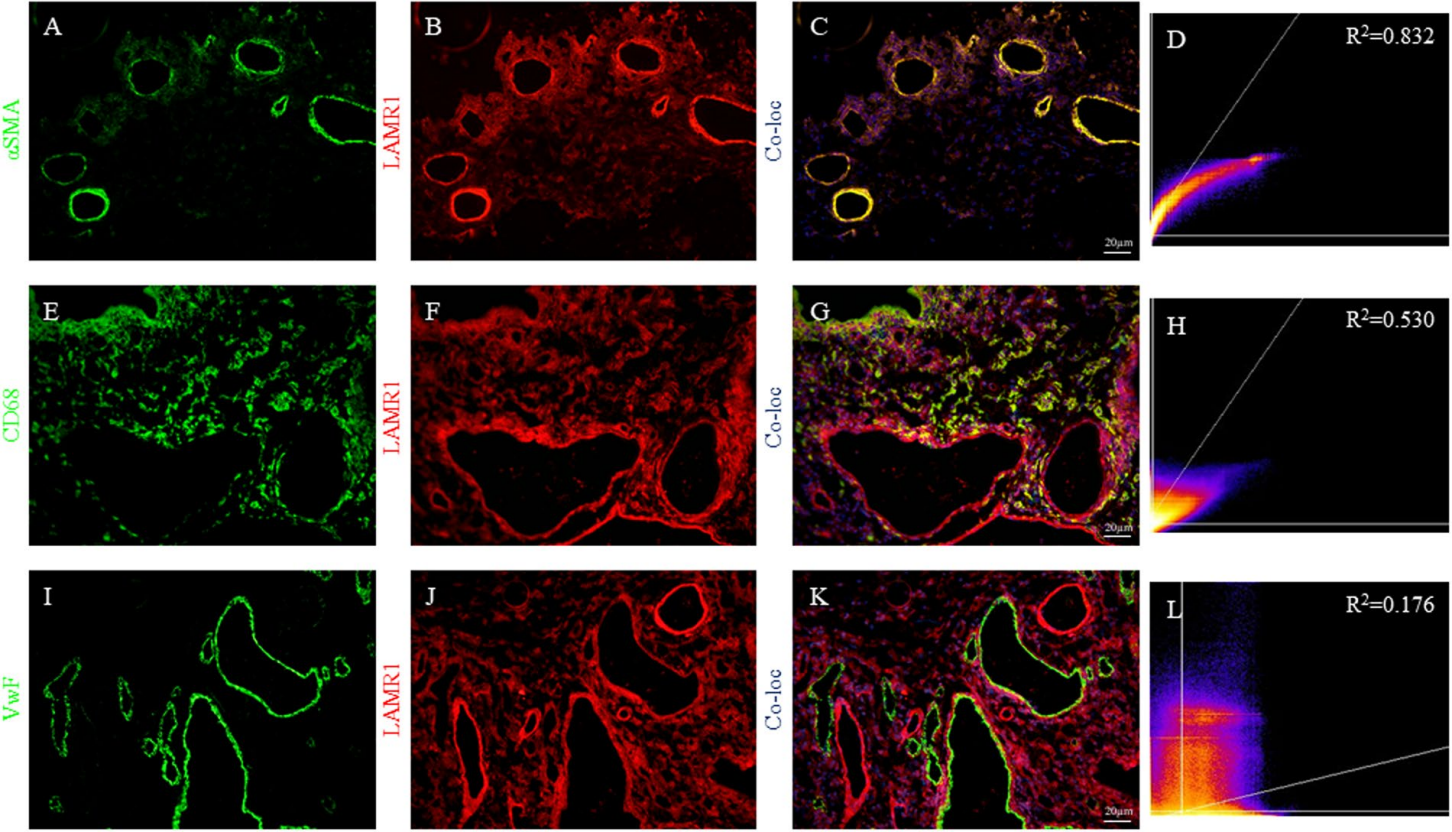
Double immunofluorescence staining of 4 µm serial frozen synovial tissue sections from a patient with RA demonstrating α-smooth muscle +ve blood vessels in green, (**A**) LAMR1 +ve cells in red (**B**) and a merged image of A, B and DAPI stained nuclei (**C**) showing strong co-localization of LAMR1 expression with smooth muscle cells of blood vessel walls. (**D**) CD68 +ve macrophages in green, (**E**) LAMR1 +ve cells in red (**F**) and a merged image of E, F and DAPI stained nuclei (**G**) showing substantial co-localization of LAMR1 expression with macrophages. (**H**) Endothelial cell staining using anti-Von Willebrand factor, (**I**) LAMR1 +ve cells in red (**J**) and a merged image of I, J and DAPI stained nuclei (**K**) demonstrating LAMR1 on blood vessels is not co-localised with endothelial cells. (**L**) All images were captured at ×250 magnification using DP73 Olympus fluorescent microscope; 2D-cytoflourogram (**D**,**H**,**L**) and co-localisation analyses performed using NIH Image J.

Synovial tissue from 20 patients with RA with a mean age of 65.7 ± 14.8 years (range 27–86 years), male to female ratio of 1:1.5 and median disease duration of 8 years (range 2–39 years) was collected irrespective of their treatments (Table 1). There were equal numbers of patients with active disease (mean DAS-28 of 4.9 ± 1.3), and inactive disease (mean DAS-28 of 1.1 ± 0.44). Tissue from 10 patients with OA (mean age 68.6 ± 9.2 years; M:F ratio of 2.3:1) and 10 normal individuals (mean age 33.9 ± 8.8 years; M:F ratio of 1.5:1) were collected as relevant controls (Table 1). Immunohistochemical staining of tissue sections using anti-LAMR1 antibody showed abundant expression of LAMR1 in synovial tissue obtained from patients with active (Fig. 7A) and inactive RA (Fig. 7B) when compared to tissue obtained from patients with OA (Fig. 7C) or control individuals (Fig. 7D). Semi-quantitation of immuno-reactive cells showed significantly higher number of LAMR1 positive cells in patients with RA when compared to OA or control individuals (Fig. 7E). Although there were measurably higher numbers of LAMR1 positive cells in synovia of patients with inactive RA when compared with those with active disease, this was not statistically significant (Fig. 7E). Similarly, there was no significant difference in LAMR1 +ve cells between controls and patients with OA, despite patients with OA having marginally higher numbers (Fig. 7E). LAMR1 was also constitutively expressed in small and medium size blood vessels in all patients and control individuals (Fig. 7F). Double immunofluorescence staining of synovial tissue of selected patients with RA showed that LAMR1 was abundantly expressed by α smooth muscle cells of small and medium sized blood vessels (Fig. 8A–D) and CD68+ macrophages (Fig. 8E–H). By contrast, there was little or no expression of LAMR1 on endothelial cells (Fig. 8I–L), T cells and/or B cells (not shown). Interestingly, the proportions of LAMR1 +ve vessels cells calculated as $\frac{(LAMR1+ve\,vessels)\,}{(LAMR1+ve+LAMR1-ve\,vessels)}\times 100$ were comparable among the different groups with 92%, 95%, 94% and 97% +ve vessels in control individuals, patients with OA, patients with active RA and patients with inactive RA respectively.

**Table 1:** Summary of synovial tissue donors at the time of sample collection.

Inactive RA	Age	Sex	Treatment	ACPA	RF	DAS
IRA1	58	M	SSZ	N/A	−	1.4
IRA2	67	M	IM Gold, MTX	N/A	+	1.8
IRA3	78	F	IM Gold	+	−	1.2
IRA4	75	M	MTX	N/A	−	1
IRA5	61	M	IM Gold	+	−	0.6
IRA6	75	F	MTX	+	−	0.7
IRA7	73	M	IM Gold	+	−	0.9
IRA8	60	F	IM Gold	+	−	2.6
IRA9	63	M	HCQ	N/A	−	0.6
IRA10	57	F	MTX	+	+	1.6
**Active RA**						
ARA1	77	F	IM Gold	N/A	+	5.2
ARA2	61	F	NSAIDs	+	−	8.3
ARA3	86	M	NSAIDs	N/A	−	7.7
ARA4	73	F	MTX	+	+	7.3
ARA5	76	F	NSAIDs	N/A	−	6.6
ARA6	31	F	NSAIDs	+	−	3.55
ARA7	67	F	Prednisone	+	−	4.57
ARA8	70	M	NSAIDs	+	+	5.8
ARA9	27	F	NSAIDs	N/A	−	3.75
ARA10	78	F	IV steroids	+	+	5.33
**OA**						
OA1	73	M				
OA2	71	M				
OA3	74	M				
OA4	71	M	Paracetamol			
OA5	56	M				
OA6	69	M	NSAIDs			
OA7	73	F	NSAIDs			
OA8	50	F	Paracetamol			
OA9	67	F	NSAIDs			
OA10	82	M	Paracetamol			
**Control**						
N1	35	M				
N2	25	F				
N3	35	M				
N4	50	M				
N5	24	M				
N6	37	F				
N7	46	M				
N8	25	F				
N9	32	F				
N10	30	M				

ACPA, anti-citrullinated protein antibody; CyA, cyclosporine A; DAS, disease activity score; F, female; HCQ, hydroxychloroquine; IM, intramuscular; M, male; MTX, methotrexate; NSAIDs, non-steroidal anti-inflammatory drugs; N/A, data not available; Pred, prednisone; RF, Rheumatoid factor; SSZ, sulfasalazine.

## Discussion

The 37/67-kDa LAMR1 is highly expressed in various malignancies^4,7,8^, however its expression in peripheral blood mononuclear cells in health and in context of chronic inflammation remain unknown. Here we found consititutive expression of LAMR1 on the surface of all peripheral blood monocytes and on a small subset B cells obtained from healthy individuals and patients with RA but was very little expression on T cells and NK cells, indicating cell specific expression. Importantly, LAMR1 level on peripheral blood monocytes obtained from patients with RA was significantly lower than healthy individuals. However, the mediators that caused suppression of LAMR1 expression in RA remain unknown. Limited data indicates that LAMR1 expression on malignant epithelial cells is regulated by key mediators relevant to the pathogenesis of RA including TNFα, IFNγ and laminins^28,29^. We found that treatment of peripheral blood monocytes with IFNγ caused a rapid and sustained surface upregulation of LAMR1 with little effect on mRNA expression suggesting surface mobilisation of the protein, while treatment with IL-10 led to a delayed upregulation of cell surface LAMR1 together with significant time-dependent increase in mRNA indicating induction of de-novo protein synthesis. Although the possibility of rapid surface translocation of LAMR1 to cell surface in response to specific stimuli such as laminin is consistent to previous observations^28,29^, its delayed surface upregulation in response to IL-10 is novel and functional consequences require further investigation. In contrast to the positive effects of the immune-regulatory cytokines IFNγ and IL-10, the prototypical pro-inflammatory cytokine, TNFα downregulated surface LAMR1 and mRNA expression in monocytes derived from healthy individuals but did not further decrease the low levels expressed on monocytes derived from patients. These results indicate that LAMR1 is tightly regulated by key pro-inflammatory and immune-regulatory cytokines. The upregulation of LAMR1 by the immune regulatory cytokines, IFNγ and IL-10 and downregulation by TNFα supports the proposal that LAMR1 may negatively regulate inflammation. Its decrease in response to TNFα *in vitro* is consistent with our *in vivo* observation that patients with active disease that are expected to have high levels of TNFα had lower levels of LAMR1 in their synovial tissue and peripheral blood monocytes.

Confirming LAMR1 functions in RA is considerably difficult due to its diverse extracellular and intracellular functions, lack of suffient knowledge on the nature of conversion of the 37-kDa LAMR1 protein to the higher molecular weight (67-kDa) species^4–6^ and difficulties in isolating LAMR1 +ve cells from inflammed synovial tissue. This is further complicated by lack of gene knockout mice because LAMR1 is embryologically letal^30^. We propose that constitutive high-level LAMR1expression on monocytes from healthy individuals may have immune regulatory roles that suppress excessive unregulated inflammation and its downregulation in RA may contribute to loss of this regulatory functions. In support with this notion, we discovered that decreased LAMR1 expression on monocytes of patients with RA strongly correlated with increased disease activity scores and pharmacological ligation of LAMR1 with EGCG on the surface of monocytes can inhibit LPS-induced TNFα production^16,17^. Furthermore, its ligation on cancer cells by laminin was reported to decrease phosphorylation of signalling proteins highly relevant in inflammation including extracellular signal regulated kinases (ERKs), c-Jun N-terminal kinases (JNKs) and p38 MAPKs, and these de-phosphorylation events that can be reversed by decrease in LAMR1 expression^7^. LAMR1 was also shown to negatively regulate GM-CSF signalling^24,25^ and its ligation by EGCG can downregulate the activating FcγRI in KU812 basophil cell line^31^, an observation that further support its proposed anti-inflammatory functions. Interestingly, several other activating receptors that are highly expressed by innate immune cells including the paired immunoglobulin-like receptor A^32^ and the activating leukocyte immunoglobulin-like receptors^33^ are known to use the immune tyrosine-based activation motif (ITAM)-containing common γ chain of the Fc receptors for activation signal transduction, hence LAMR1 may have broader immune regulatory effects. In this study we found that cell surface LAMR1 promoted binding of peripheral blood monocytes to laminin-1, an extracellular matrix protein produced by synovial fibroblasts and endothelial cells derived from patients with RA^22^ and this binding was significantly modulated by a key cytokine involved in the pathogenesis of RA, IFNγ. However, it is uncertain if this interaction also triggers intracellular signalling mechanisms that regulate inflammation as observed in cancer cells^7^. It is plausible that ligation of LAMR1 in synovial tissue of patients with RA by laminin-1^22^ may suppress MAPK signalling leading to decreased inflammation in patients with inactive RA that express high levels of LAMR1 as contrasted to patients with active RA that have low levels of this receptor.

Taken together, this study presented expression, regulation and functions of LAMR1 that suggest that LAMR1 expressed on a selected subsets of blood leukocytes and synovial tissue cells may play key immune regulatory role on monocytes and its downregulation my contribute to the pathogenesis of RA. Future studies are required to investigate to whether increasing LAMR1 expression and/or its suppressive functions on leukocytes may serve as a novel theraputic strategy in the treatemt of RA.

## Material and Methods

### Antibodies and reagents

A mouse anti-human LAMR1 monoclonal antibody from Lifespan (Seattle, WA, USA) and a mouse IgG1 control monoclonal antibody from Sigma-Aldrich were used for flow cytometry, along with CD14-FITC or CD3-FITC, CD19-Percp and CD56-APC and their corresponding fluorochrome and isotype-matched IgG controls; all purchased from BD Biosciences (San Jose, CA, USA). Phycoerythrin-conjugated goat anti-mouse secondary antibody was acquired from Jackson Immunoresearch (West Grove, PA, USA). Azide-free anti-human LAMR1 monoclonal antibody (Lifespan) was used to block *in vitro* adhesion of monocytes to laminin 1. Purified laminin-1 from mouse Engelbreth-Holm-Swarm tumor cells **(**11243217001 ROCHE) was purchased from Sigma-Aldrich (Castle Hill, NSW, Australia). A rabbit anti-human LAMR1 Ab (Genetex, Irvine, CA, USA) and a corresponding control rabbit IgG (Dako, Glostrup, Denmark) were used for immunostaining and Western blotting. Mouse monoclonal antibodies against human macrophages (CD68) and endothelial cells (von Willebrand factor), and mouse monoclonal antibodies against α-smooth muscle actin were purchased from Dako and Sigma-Aldrich (St Louis, MO, USA) respectively. Normal goat serum, biotinylated goat anti-mouse or goat anti-rabbit secondary antibodies, avidin biotin-alkaline phosphatase complex (Vectastain kit), and alkaline phosphatase substrate (Vector Red) were from Vector Laboratories (Burlingame, CA, USA). Alexa-488 conjugated goat anti-mouse and Alexa-568 conjugated goat anti-rabbit was purchased from Molecular probes (Eugene, OR, USA).

### Peripheral blood and synovial tissue from patients and controls

Peripheral blood in acid citrate dextrose anti-coagulant was collected from a cohort of 22 patients with established RA (median disease duration of 6.7 years), and 25 age and sex-matched healthy controls. All patients with RA fulfilled the 1987 ACR criteria for RA^34^. Clinical assessments of RA patients included modified health assessment questionnaire^35^, DAS28 (Disease Activity Score assessing 28 joints for pain and tenderness, c-reactive protein (CRP) (>20 mg/L) and visual analog scores for disease activity (measured on a 10-cm horizontal scale anchored at both ends)^36^. Inactive disease was defined as a DAS-28 CRP score of ≤2.6 as defined by EULAR criteria^37^. All patients were under various biologic and/or non-biologic disease modifying antirheumatic drug treatments for at least 6 months at the time of blood collection. Archival formalin-fixed, paraffin-embedded synovial tissue biopsies from a different cohort of patients were generously supplied by Professor M Smith, Flinders University, Adelaide, Australia. These include 20 from patients with RA, 10 from patients with OA, and 10 normal synovial tissues from individuals undergoing arthroscopic surgery for acute traumatic meniscus rapture. The Ethics Committee for South Western Sydney Local Health District approved the study on the peripheral blood of patients with RA and control individuals, and informed consent was obtained from all participants (HREC11/LPOOL/71). Buffy coats from healthy individuals were obtained from Australian Red Cross Blood Service under the material supply agreement number 11-09NSW-16. All the experiments were performed in accordance with the approved protocols and the study was conducted in line with the Helsinki Declaration.

### Isolation of peripheral blood mononuclear cells and *in-vitro* culture

Peripheral blood mononuclear cells were isolated by gradient centrifugation as described^38^. In brief, blood from healthy donors or patients with rheumatoid arthritis was diluted 1:1 in phosphate-buffered saline (PBS) and overlaid on Lymphoprep (Stem cell Technologies, Tullamarine, Victoria, Australia). Following density gradient separation PBMCs were collected and washed twice in sterile PBS and total and differential count performed using the AcT diff Analyzer (Beckman-Coulter). Cells were then immediately used for flow cytometry or treated with relevant recombinant cytokines *in vitro* for 0–18 h. All PBMCs were prepared and cultured under strict LPS-minimised conditions by using LPS-free culture media and reagents, z-filtration of buffers (<0.05 endotoxin units/ml), and by heat and/or chemical treatment of glassware and fume hoods (<0.2 units/item, Techni Tool, PA, USA).

### Flow cytometric analysis of LAMR1 expression on the surface of peripheral blood monocytes

Freshly isolated PBMCs from controls and patients were washed once in PBS containing 0.05% NaN_3_ and 0.1% BSA (FACS buffer) before incubating two tubes containing mouse monoclonal IgG anti-LAMR1 or a tube with mouse IgG at 5 µg/ml for 45 min at room temperature. Tubes were washed twice in FACS buffer, stained with goat anti-mouse PE for 45 min at 4 °C, washed twice. This was followed by further incubation of the cells stained for LAMR1 with mouse monoclonal IgG against CD14-FITC or a combination of CD3-FITC, CD19-Percp and CD56-APC and cells stained with negative control IgG incubated with FITC, Percp and APC-conjugated negative control mAbs for 20 min at room temperature. Following further two washes, cells were fixed with 1% paraformaldehyde and data acquired using FACS Calibur flow cytometer (BD Biosciences). A total 20,000 events for each tube were acquired and surface expression of LAMR1 in monocyte and lymphocyte gates analysed using FlowJo software (Tree star Inc.).

To assess the effects of IFNγ, TNFα, IL-1β or IL-10 to surface expression of LAMR1 on monocytes, PBMCs from 5 healthy individuals and 5 patients with active disease (DAS ≥ 2.6) were resuspended at a concentration of 1–2×10^6^/mL in RPMI 1640 (Sigma-Aldrich) supplemented with 10% foetal bovine serum (FBS), 1% MEM sodium pyruvate, 1% MEM non-essential amino acids solution (all from Invitrogen), 2 mM L-glutamine, 100 U/mL penicillin and 100 μg/mL streptomycin (all from Sigma-Aldrich, St Louis, MO) and cultured with/without recombinant TNFα (25 ng/mL), IFNγ (50 ng/mL), IL-1β (25 ng/mL) or IL-10 (10 ng/mL), all purchased from R&D Systems (Minneapolis, MN, USA). Cells were cultured in sterile 15 mL Falcon tubes (BD Biosciences) at 37 °C, 5% CO_2_ incubator for 4, 12 or 18 h, washed in PBS and used for flow cytometry as described above.

### Quantitative Real Time PCR for LAMR1 mRNA in primary monocytes of healthy individuals

Primary peripheral blood monocytes were negatively selected from PBMCs of 5 healthy donors (60 ml of blood) using Miltenyi magnetic bead isolation kit for monocyte as previously described^38^. These freshly purified monocytes (95–99% pure) were incubated at 1 × 10^6^/ml with or without recombinant TNFα (25 ng/mL), IFNγ (50 ng/mL), IL-1β (25 ng/mL) or IL-10 (10 ng/mL), for 2, 6 or 12 h in 48-well flat bottom Costar plates. Cells were then pelleted by centrifugation at 300RCF for 5 min, resuspended in 300uL TRIzol and total RNA extracted according the manufacturer’s instructions (Life Technologies, VIC, Australia). Reverse transcription was performed on 0.4–0.5 µg DNase treated RNA using SuperScript III Kit (Invitrogen) in a final volume of 20 µl. Aliquots (1 µl) of cDNA, 1.25 µM of forward and reverse primer set each and SYBR Green I qPCR Master mix (Invitrogen) were used for qPCR in triplicates utilising the LightCycler 480 Real-Time PCR System (Roche Applied Science, IN, USA) according to the following conditions: 95 °C for 5 min for initialisation, then 40 cycles of PCR (each cycle 95 °C, 10 seconds; 62 °C, 10 seconds; 72 °C, 10 seconds). Human β-actin primers were purchased from Invitrogen (Forward 5′-CATGTACGTTGCTATCCAGGC and Reverse 5′ TAGCACGCACTGTAATTCCTC) and LAMR1 primers were purchased from Sigma (Forward 5′-GGAGGAAUUUCAGGGUGAA-3′ and Reverse 5′-UUCACCCUGAAAUUCCUCC-3′). Average Threshold-cycle (CT) values for LAMR1 were normalised by dividing values to the corresponding average CT of the housekeeping gene β-actin and data presented as fold change from LAMR1 of untreated (control) cells for each timepoint^33^.

### *In vitro* adhesion of primary monocytes to laminin-1

Freshly purified monocytes (see above) from 3 healthy individuals and 3 patients with active RA was used for adhesion assay in triplicate wells using a Real-time Cell Analyzer xCELLigence system (Roche Applied Science, Dee Why, NSW, Australia)^39^. In brief, 96-well ‘E-plates’, were coated with 100 µl of 50 μg/ml laminin-1, incubated at 4 °C overnight, then blocked with 1% (w/v) bovine serum albumin/PBS for 2 h. Magnetic bead isolated monocytes in PBS were pre-incubated with 10 μg/ml of azide-free anti-LAMR1 mouse IgG_1_ (Lifespan) or 10 μg/ml of isotype-matched irrelevant mouse IgG control (Sigma) for 15 min at room temperature, washed once with cold PBS and re-suspended in 15 ml round bottom Falcon tubes at 5 × 10^6^ cell/ml in 1% (w/v) bovine serum albumin/RPMI 1640 with or without TNFα (25 ng/ml) or IFNγ (50 ng/ml). After a 4-hour incubation in humidified 37 °C incubator at 5% CO_2_ air, cells were transferred to E-plate wells 1 × 10^5^ cells/well (100 µl per well). Plates were then placed inside humidified 37 °C incubator at 5% CO_2_ air, and electrodes embedded in each well measured cell adhesion based upon impedance, represented as cell index (CI) that were measured every 1 min for 3 h. Triplicate wells coated with 1% BSA in PBS alone were used as controls for background adhesion. For antibody blocking experiments, purified monocytes in PBS were pre-incubated with 10 μg/ml of azide-free anti-LAMR1 mouse IgG_1_ (Lifespan) or 10 μg/ml of isotype-matched irrelevant mouse IgG control (Sigma) for 15 min at room temperature, washed once with cold PBS and re-suspended in 15 ml round bottom Falcon tubes at 5 × 10^6^ cell/ml in 1% (w/v) bovine serum albumin/RPMI 1640 with or without IFNγ, TNFα, IL-1β or IL-10 and used for adhesion assay as described above.

### Immunohistochemical staining of synovial tissue

Paraffin-embedded synovial sections were cut at 2–4 µm onto silanized slides, dewaxed in xylene and rehydrated by immersion in diminishing concentrations of ethanol. They were then washed in Tris-buffered saline (TBS), blocked in 20% serum in TBS at room temperature for 20 min and stained with 6.2 µg/mL of rabbit anti-human LAMR1 primary antibody overnight at 4 °C (diluted in TBS/2% BSA). The following day slides were washed three times with TBS and incubated with 0.5 µg/mL of biotinylated goat anti-rabbit IgG (Dako) for 2 h at room temperature, washed three times, and incubated with streptavidin-alkaline phosphatase conjugate (Vectastain AP, Vector Labs) according to the manufacturer’s instructions for 45 min at room temperature. The slides were washed three times in TBS, washed once in 0.1 M TRIS pH8.2 for 5 min and finally treated with alkaline phosphatase substrate (Vector Red, Vector Labs) at room temperature for 10–20 min. They were then counterstained with haematoxylin, dehydrated by exposure to increasing concentrations of ethanol and xylene, and mounted.

To determine the cellular sources of LAMR1 in synovial tissue of patients with RA, 6–8 µm frozen tissue sections from 5 patients were used for double immunofluorescence staining. In brief, tissue sections on silanized slides were fixed in acetone for 10 min, air dried, equilibrated with TBS and blocked with 20% goat serum in TBS for 20 min at room temperature. This was followed by incubation of slides with either rabbit anti-LAMR1 antibody or irrelevant rabbit control IgG (10 µg/ml each diluted in TBS+2% BSA) at 4 °C overnight. After three TBS washes slides were stained with 2 µg/ml TBS+2% BSA Alexa-568 conjugated goat anti-rabbit IgG at room temperature for 2 h and thoroughly washed with TBS. Slides were then co-stained with mouse anti-CD68 (1 µg/ml), anti- von Willebrand factor (vWF) or anti-α smooth muscle (both 0.5 µg/ml) monoclonal antibodies for 2 h at room temperature followed by washes (4 times with TBS) and incubation with Alex-488 conjugated goat anti-mouse IgG for 1 h. Finally, sections were washed and mounted with DAPI containing mounting media (Molecular Probes) and visualised and imaged using DP73 Olympus fluorescent microscope (Olympus, Mount Waverly, Victoria, Australia). The extent of co-localisation between vascular smooth muscle cells, macrophages or endothelial cells was analysed using NIH Image J, version 1.52.

### Semi-quantitation of immunohistochemical staining

Following immunohistochemical staining, the number of LAMR1-positive cells and blood vessels in synovial tissue sections was evaluated by counting immunoreactive cells/vessels in contiguous fields across the whole section as described previously^40^. In brief, after ensuring that sections stained with isotype control exhibited no significant immunoreactivity, numbers of positive cells and vessels in each field (×250 magnification) were enumerated. Although the numbers of fields varied from patient to patient (range 12–33 fields), and there were obvious regional variations in staining, the median count per field for each subject and mean ± SEM per group is reported as a conservative measure of the staining for each antibody. A standard haematoxylin and eosin stain was used to determine the extent of inflammatory cell infiltration and the numbers of small blood vessels in tissue sections. All sections were counted by an independent observer. Immunostaining for LAMR1 using multiple sections of tissue from individual patients was highly reproducible.

### Statistical analysis

Statistical analysis was performed using GraphPad Prism Version 6 (GraphPad Software Inc, La Jolla, CA, USA). Unpaired t test with Welch’s correction was used to compare differences in mean fluorescence intensities of LAMR1 on monocytes or proportions of B cells in all patients with RA and healthy individuals; patients with active versus inactive disease; patients with active disease versus healthy individuals and patients with inactive disease versus healthy individuals. Linear regression analysis and Spearman correlations were used to examine the relationship between LAMR1 expression on monocytes and DAS28 scores of RA individuals. Unpaired t-test was used to compare the magnitude of cytokine effects on the expression of LAMR1 on monocytes from healthy controls and patients with RA. Changes in LAMR1 mRNA or protein (MFI) in monocytes after cytokine treatment overtime was compared to corresponding media alone control samples using one-way ANOVA with Dunnett’s correction. Adhesion of cytokine-treated monocytes ± blocking antibody to laminin-1 overtime was compared to corresponding media alone control samples ± negative control antibody, using one-way ANOVA with Dunnett’s correction for multiple comparisons. Mean counts ± SEM of LAMR1 +ve cells or vessels in synovial tissue in patients and controls were calculated from the median values of the whole tissue section of everyone in each group, and statistical significance among each group determined using one-way ANOVA with Dunn’s correction.
